# Associations of Symptoms of ADHD and Oppositional Defiant Disorder (ODD) in Adolescence With Occupational Outcomes and Incomes in Adulthood

**DOI:** 10.1177/10870547241259329

**Published:** 2024-06-12

**Authors:** Sampo Seppä, Sanna Huikari, Marko Korhonen, Tanja Nordström, Tuula Hurtig, Anu-Helmi Halt

**Affiliations:** 1 Research Unit of Clinical Medicine, University of Oulu, Oulu, Finland; 2 Department of Economics, Accounting and Finance, University of Oulu, Oulu, Finland; 3 Research Unit of Population Health, University of Oulu, Oulu, Finland; 4 Clinic of Child Psychiatry, Oulu University Hospital, Oulu, Finland; 5 Department of Psychiatry, Oulu University Hospital, Oulu, Finland

**Keywords:** ADHD, oppositional defiant disorder, SWAN, occupational outcome

## Abstract

**Objective:**

The purpose of this study was to examine the associations of ADHD and ODD symptoms in adolescence with occupational outcomes and incomes in adulthood within the Northern Finland Birth Cohort 1986 (NFBC1986).

**Method:**

ADHD symptoms were evaluated at ages 15 to 16 years using the Strengths and Weaknesses of ADHD symptoms and Normal Behaviors (SWAN) scale. ODD symptoms were assessed using a 7-point scale, like the SWAN assessment.

**Results:**

Symptoms of ADHD and ADHD + ODD were associated with elevated rates of unemployment, increased sick days, and lower annual incomes compared to controls for both sexes. Symptoms of ODD were associated with higher unemployment and more sick days for males, although these associations did not reach statistical significance in their association with annual incomes.

**Conclusion:**

Symptoms of ADHD were associated with adverse occupational outcomes and lower incomes. Furthermore, symptoms of ODD were associated with occupational outcomes but not with incomes.

## Introduction

ADHD and Oppositional Defiant Disorder (ODD) are common psychiatric disorders (Knappe et al., 2022) and highly comorbid with each other. ODD is estimated to co-occur with ADHD in approximately 50% to 60% of population-based samples (Kessler et al., 2014; Reale et al., 2017). ADHD involves symptoms of inattention, hyperactivity, and impulsivity (American Psychiatric Association, 2013), affecting around 5% of children (Polanczyk et al., 2007) and 2.5% of adults worldwide (Simon et al., 2009). According to DSM-5, ADHD symptoms are defined by their impact on reducing the quality of social, academic, or occupational functioning (American Psychiatric Association, 2013). ODD symptoms involve a pattern of angry or an irritable mood, vindictiveness toward others, and argumentative or defiant behavior (American Psychiatric Association, 2013). The estimated global prevalence of ODD among children aged 18 years or younger is 3.3% (Canino et al., 2010).

Symptoms of ADHD have been associated with adverse occupational outcomes, including high unemployment (Jangmo et al., 2021; Küpper et al., 2012), an increased likelihood of receiving a work disability pension (Erskine et al., 2016; Halmøy et al., 2009), and a higher probability of changing jobs (Ahlberg et al., 2023; Barkley et al., 2006). Furthermore, symptoms of ADHD are linked to lower earnings. In a longitudinal study comparing a “never ADHD” group (*n* = 13,710) to an “ever ADHD” group (*n* = 726) from a national US sample, the latter group experienced an annual earning reduction of approximately 33% (Fletcher, 2014). A Swedish longitudinal study, based on a large population-based sample, revealed that individuals with ADHD (*n* = 28,914) had, on average, 17% lower income annually compared to the control group (*n* = 1,167,830; Jangmo et al., 2021). Additionally, a British national cohort study showed that attention deficit problems at age 10 were significantly associated with lower earnings at age 30 for male and female workers. A male worker at the 95th percentile on the attention deficit index at age 10 was estimated to earn around 9% less than a male in the control group, while for females, the comparable figure was 20% (Knapp et al., 2011).

Nevertheless, some studies have not found an association between ADHD symptoms and income levels (Barkley et al., 2006; Roy et al., 2017). More research on ADHD symptoms and their impact on occupational outcomes is needed. It is essential to note that many of the current population-based studies have not considered possible comorbid psychiatric conditions associated with ADHD. Additionally, a significant number of studies rely on self-reports of ADHD symptoms or occupational outcomes (Fletcher, 2014; Knapp et al., 2011; Roy et al., 2017).

Although ODD has conventionally been considered a disorder exclusive to childhood and adolescence, symptoms of ODD continue to be reported into young adulthood (B. Leadbeater et al., 2012). Functional outcomes associated with ODD symptoms in adolescence include family conflicts, impaired interpersonal relationships, peer rejection, and academic challenges (Burke et al., 2014; Seppä et al., 2023). Limited research has explored the associations of ODD symptoms in adolescence with subsequent educational or occupational functioning in adulthood. A previous study from the Northern Finland Birth Cohort 1986 showed that ODD symptoms in adolescent females predicted lower educational attainment in adulthood compared to controls (Seppä et al., 2023). Furthermore, a population-based study by Leadbeater et al. suggested that ODD symptoms in adolescence were associated with lower educational attainment for males in young adulthood. However, ODD symptoms were not associated with occupational outcomes such as employment status, the number of jobs, and hours worked. This lack of association may be due to other factors influencing employment, including minimum wage standards and engagement in part-time and youth-oriented jobs (B. J. Leadbeater & Ames, 2017). The results of a 20-year follow-up study suggested that irritable symptoms of ODD in adolescence were linked to lower income and educational attainment in adulthood. This relationship was not mediated by major depression, generalized anxiety disorder, or dysthymia at follow-up (Stringaris et al., 2009). The symptoms of irritability and defiance characterizing ODD can potentially lead to problematic behaviors during interactions with authorities, coworkers, and customers in academic or occupational settings.

To the best of our knowledge, this is the first study to include symptomatic groups of ADHD, ODD, and ADHD + ODD, along with a control group for both sexes, to examine their associations with occupational outcomes and incomes. The purpose is to study longitudinal associations from adolescence to adulthood using a large, unselected population-based sample: the Northern Finland Birth Cohort 1986 (NFBC 1986). The data used in this study provide the opportunity to consider several potential confounders, such as the educational level of the participants’ parents, the participants’ own educational level, family type, and psychiatric disorders other than ADHD or ODD. Occupational outcome and income data are based on a nationwide register held by the Finnish Centre for Pensions, ensuring reliable information, and a low attrition rate.

We aimed to investigate: (1) whether symptoms of ADHD in adolescence are associated with occupational outcomes and incomes in adulthood, (2) whether symptoms of ODD in adolescence are associated with occupational outcomes and incomes in adulthood and (3) whether comorbid symptoms of ADHD and ODD are associated with occupational outcomes and incomes in adulthood.

## Methods

This population-based follow-up study utilizes the NFBC1986 as its study sample. The original cohort consisted of 9,432 children whose expected date of birth ranged from July 1, 1985, to June 30, 1986, encompassing 99% of all children born alive in the target period in the two northernmost provinces of Finland. Further details on the NFBC1986 data collection and cohort design are available in previous reports (Järvelin et al., 1993; University of Oulu, 1986).

When the participants were 15 to 16 years old (*n* = 9,215), their parents received questionnaires that included The Strengths and Weaknesses of ADHD-Symptoms and Normal-Behaviors (SWAN) rating scale for ADHD symptoms (J. M. Swanson et al., 2012). A total of 6,985 of these participants’ parents (75.8%) returned the questionnaire together with a statement of their informed consent. The Ethical Committee of the Northern Ostrobothnia Hospital District approved the study (Cohort 1986: Northern Ostrobothnia Hospital District Ethical Committee 108/2017 (15.1.2018)).

### Exposure Variables: Symptoms of ADHD and ODD

The Strengths and Weaknesses of ADHD Symptoms and Normal Behaviors (SWAN) scale is a revised version of the SNAP-IV, which was developed by J. M. Swanson et al. (2012). This scale measures symptoms in attention, hyperactivity/impulsivity, and disruptive behavior, comprising a total of 30 items (J. M. Swanson et al., 2012). Parents rated ADHD symptoms based on the first 18 items of the SWAN scale, starting from “Give close attention to detail and avoid careless mistakes” and extending to “Enter into conversations and games (control interrupting/intruding).” These items correspond to the ADHD symptoms described in the DSM-IV-TR (American Psychiatric Association, 2000). The SWAN scores were used to identify ADHD symptoms by requiring SWAN scores to exceed the 95th percentile on either Inattentive scale (items 1–9) or Hyperactive-Impulsive scale (items 10–18).

The SWAN scale was supplemented with ODD symptoms based on eight items corresponding to the ODD symptom criteria listed in the DSM-IV-TR (American Psychiatric Association, 2000). The items included “Control temper,” “Avoid arguing with adults,” “Follow adult requests or rules (follow directions),” “Avoid deliberately doing things that annoy others,” “Assume responsibility for mistakes or misbehavior,” “Ignore annoyances of others,” “Control anger and resentment,” and “Control spitefulness and vindictiveness.”

Both ADHD and ODD symptoms, with their respective items, were assessed on a seven-point scale anchored to average behavior, where “Far Below Average” = 3, “Below Average” = 2, “Somewhat Below Average” = 1, “Average” = 0, “Somewhat Above Average” = −1, “Above Average” = −2, and “Far Above Average” = −3. This scale resulted in normally distributed behavioral traits. The 95th percentile of the distribution was used as the cut-off point for identifying problems, in accordance with the scale developer’s recommendation (J. Swanson et al., 2001) and consistent with previous studies (Smalley et al., 2007). Using this approach, four groups were created: (1) those with ADHD symptoms, (2) those with ODD symptoms, (3) those with ADHD + ODD symptoms, and (4) community controls, who exhibited neither ADHD nor ODD symptoms.

The SWAN scale has demonstrated good validity and reliability, comparable to that found in other scales such as Conners’ Continuous Performance Test (CPT-II) and the Disruptive Behavior Rating Scale (DBRS; Arnett et al., 2013; Cornish et al., 2005). In the study by Arnett et al., the test-retest reliability estimate for the SWAN ranged from .72 to .90, with a mean value of .82.

### ADHD Symptom Clusters

ADHD symptom clusters were studied as additional analyses for this study. DSM-5 lists three presentations of ADHD: predominantly Inattentive, Hyperactive-Impulsive, and Combined (Inattentive + Hyperactive-Impulsive, (American Psychiatric Association, 2013). Considering the instability of ADHD symptom presentation over time and the complex issue of informant effects (Nigg et al., 2010), we chose to analyze the continuous measures of the inattentive, hyperactive-impulsive, and combined symptom clusters rather than categorizing ADHD into discrete subtypes. Inattentive symptoms were assessed based on the first nine items of the SWAN scale, starting from “Give close attention to detail and avoid careless mistakes” and extending to “Remember daily activities.” Hyperactive-Impulsive symptoms were assessed based on the items 10 to 18 on the SWAN scale, starting from “Sit still (control movement of hands/feet or control squirming)” and extending to “Enter into conversations and games (control interrupting/intruding).” Combined symptoms were based on both of those scales.

### Outcome Variables: Occupational Outcomes and Incomes

To obtain individual-level data on the gross income of cohort members, we used the register of the Finnish Tax Administration (https://www.vero.fi/en). The annual gross income data were available from 2006 to 2016. In our analysis, we used income information from the years 2006 and 2016. The annual incomes are presented in 2016 euros; that is, income in the year 2006 was adjusted to 2016 using the Consumer Price Index (CPI) as a converter (Statistics Finland, 2023a, 2023c).

Experienced unemployment was measured as the total number of unemployment days per participant during two separate timespans, with data retrieved from the Finnish Centre for Pensions (https://www.etk.fi/en/), spanning from 2006 to 2019. Additionally, data on sick leave days were obtained from the register of the Social Insurance Institution and the Finnish Center for Pensions. Permanent residents of Finland are registered for social security benefits, including sickness insurance from the Social Insurance Institution, and employment-related data and benefits from the Finnish Center of Pensions. The data are available to all workers and included the number of days of sick leave longer than 10 days. Sick leaves lasting 10 days or less were not present in the registers, as this period is not covered by the sickness allowance under social insurance. Detailed information on the healthcare registers and sickness allowance systems in Finland is provided elsewhere (Rissanen et al., 2021).

We examined sick and unemployed days in two distinct time spans: 2006 to 2010 and 2011 to 2019. This division was chosen to, at least partially, separately examine the period when most individuals would be in the workforce, that is, after graduating (OECD, 2011). In the initial period under examination, the participants were 20 to 24 years old, while in the latter period, they were 25 to 33 years old. Additionally, for the entire population born in 1986 in Finland, the employment rate exceeds 70% at the age of 25, specifically in the year 2011 (Statistics Finland, 2023b).

### Covariates

Numerous risk factors influencing occupational outcomes and incomes have been documented in the literature, and many of these variables have been associated with ADHD and ODD (Jangmo et al., 2021; Knapp et al., 2011; Vergunst et al., 2019). To elucidate the connections between ADHD and ODD symptoms in adolescence and subsequent occupational outcomes and incomes in adulthood, we adjusted the regression analyses with the following covariates: the educational level of the participants’ parents, the participants’ own educational level, the participants’ family type (distinguishing between those with both biological parents and other family structures), and the presence of psychiatric disorders other than ADHD or ODD (categorized as yes or no).

The educational level of the participants’ parents was derived from questionnaire data, while the participants’ highest education levels were extracted from registry information. Both were classified into three categories (comprehensive school, secondary education, higher education). Information on family type, categorized as “family with two biological parents (=1),” and “other (=0),” was based on questionnaire data. Data on psychiatric disorders other than ADHD or ODD were sourced from the Care Register for Health Care maintained by the Finnish Institute for Health and Welfare. This included information on specialized care inpatient (until 2016) and outpatient treatments (1998–2016) as well as primary care outpatient treatments (2011–2016), classified as yes (=1) or no (=0). Participants solely diagnosed with ADHD or ODD in health notifications were not considered in connection with this variable.

### Statistical Methods

The Pearson χ^2^ test was employed to assess the independence of means for contingency tables in analyzing categorical variables. For continuous variables (means of unemployment days and sick days), analysis of variance (ANOVA) was conducted, along with the Welch test where applicable. Additionally, post hoc tests for continuous variables were performed using the Tukey test and the Games-Howell test where appropriate. To examine the association between annual income and the ADHD, ODD, and ADHD + ODD groups, linear OLS regression with dependent variable in natural logarithmic form was employed. It is common in labor economics to use the natural logarithm transformation of income because the income distribution tends to be highly skewed (Gujarati, 2011, p. 53). Also, since logarithms convert changes in variables into percentage changes, income gaps across different groups are naturally expressed in percentages terms (Stock & Watson, 2020, p. 289). All the statistical analyses were carried out using Stata 16.0 (StataCorp LLC). All the tests were two-tailed, and all *p*-values less than .05 were considered as statistically significant.

## Results

### Characteristics of the Study Population

The characteristics of the study population are presented in Table 1, revealing that 66.7% of adolescents with symptoms of ADHD were males, while 45.3% of those in the ODD group were males (see Table 1). In the control group, 51.3% of the participants had attained higher education (university or university of applied sciences) by the age of 32, whereas for the ADHD + ODD group, this figure was 14.7%. In the control group, 19.9% of the participants’ parents had attained an institution of higher education as their highest educational level, whereas in the ADHD + ODD group, this figure was 10.1%. Concerning the family type of the participants, 78.6% of adolescents in the control group lived with both of their biological parents, while this was the case for 64.9% of adolescents in the ADHD + ODD group. The ADHD + ODD group exhibited the highest prevalence of other psychiatric disorders in the health notifications (43.7%), exceeding those associated with the other study groups.

**Table 1.** Descriptive Statistics of the Study Population.

	ADHD, *n* (%)	ODD, *n* (%)	ADHD + ODD, *n* (%)	Controls, *n* (%)	χ^2^ (*df*), *p*-value	Post hoc test^a^ (T = Tukey, G = Games-Howell)
Sex of participants					48.2 (3), *p* < .001	A&O/AO/C, AO&A/C *(G)*
Males	240 (66.7)	63 (45.3)	114 (57.3)	2,704 (48.9)		
Females	120 (33.3)	77 (54.7)	85 (42.7)	2,828 (51.1)		
Total	360 (100)	139 (100)	199 (100)	5,532 (100)		
Educational attainments of the participants at age 32 years					280.5 (6), *p* < .001	A&O/AO/C, AO&A/C, O&C *(G)*
Comprehensive school	37 (10.4)	13 (9.4)	35 (17.8)	232 (4.2)		
Upper secondary school	249 (70.1)	69 (49.6)	133 (67.5)	2,416 (44.5)		
Institution of higher education	69 (19.4)	57 (41.0)	29 (14.7)	2,834 (51.3)		
Educational attainments of the participants’ parents					23.2 (6), *p* < .001	A&C, C&O/AO *(T)*
Comprehensive school	94 (26.8)	34 (25.0)	44 (23.4)	1,259 (23.3)		
Upper secondary school	213 (60.7)	75 (55.2)	125 (66.5)	3,076 (56.9)		
Institution of higher education	44 (12.5)	27 (19.9)	19 (10.1)	1,075 (19.9)		
Family type of the participants					49.7 (3), *p* < .001	A&C, C&O/AO *(G)*
Both parents	230 (67.7)	88 (65.7)	120 (64.9)	4,190 (78.6)		
Others	110 (32.4)	46 (34.3)	65 (35.1)	1,141 (21.4)		
Psychiatric disorders of the participants^b^					110.0 (3), *p* < .001	A&AO/C, C&O/AO *(G)*
Yes	110 (30.6)	47 (33.8)	87 (43.7)	1,049 (19.0)		
No	250 (69.4)	92 (66.2)	112 (56.3)	4,483 (81.0)		

a Pairwise crosstabulations between the study groups; only significant differences (*p* < .05) are noted. A = ADHD; O = ODD; AO = ADHD + ODD; C = controls. (T = Tukey, G = Games-Howell).

b Other than ADHD or ODD (yes or no).

### Number of Days of Unemployment Within the Study Groups

The number of days that participants were unemployed is presented in Table 2. Among males, all symptomatic groups had more days of unemployment between the ages of 25 and 33 years (2011–2019) compared to the control group (*p* < .05). Among females, this was the case for the ADHD and ADHD + ODD groups (*p* < .05). The female ODD group also experienced more days of unemployment (477) than the control group (350) during that period, but the difference did not reach statistical significance in post hoc comparisons.

**Table 2.** Days^b^ of Unemployment at Ages 20 to 24 Years (2006–2010) and 25 to 33 Years (2011–2019) in the Study Groups, Males, and Females.

	ADHD	ODD	ADHD + ODD	Controls	Anova/Welch	Post hoc test^a^ (T = Tukey, G = Games-Howell)
	*M* (*SD*)	*M* (*SD*)	*M* (*SD*)	*M* (*SD*)		
Males						
2006–2010	260 (348)	208 (311)	276 (356)	145 (265)	*p* < .001	C < A, C < AO *(G)*
2011–2019	567 (813)	550 (732)	589 (838)	334 (624)	*p* < .001	C < A, C < O, C < AO *(G)*
Females						
2006–2010	204 (263)	182 (265)	196 (279)	135 (251)	*p* = .002	C < A *(G)*
2011–2019	577 (775)	477 (606)	621 (751)	350 (552)	*p* < .001	C < A, C < AO *(G)*

*Note*. A = ADHD; O = ODD; AO = ADHD + ODD; C = controls. (T = Tukey, G = Games-Howell).

a Only significant differences (*p* < .05) are noted.

b Calendar days.

### Number of Sick Days Within the Study Groups

The number of sick days is presented in Table 3. Among males, all symptomatic groups had more sick days between the ages of 25 and 33 years than the control group (*p* < .05). Among females, all symptomatic groups had more sick days than the control group in young adulthood, between the ages of 20 and 24 years (*p* < .05).

**Table 3.** Sick Days^b^ at Ages 20 to 24 Years (2006–2010) and 25 to 33 Years (2011–2019), for Males and Females.

	ADHD	ODD	ADHD + ODD	Controls	Anova/Welch	post hoc test^a^ (T = Tukey, G = Games-Howell)
	*M* (*SD*)	*M* (*SD*)	*M* (*SD*)	*M* (*SD*)		
Males						
2006–2010	10 (45)	13 (52)	20 (73)	6 (32)	*p* < .001	C < AO *(G)*
2011–2019	33 (83)	42 (117)	39 (101)	18 (65)	*p* < .001	C < A, C < O, C < AO *(G)*
Females						
2006–2010	30 (85)	4 (17)	17 (47)	6 (31)	*p* < .001	C < A, C < AO, O < A *(G)*
2011–2019	51 (120)	46 (98)	46 (114)	25 (81)	*p* < .001	C < A *(G)*

*Note*. A = ADHD; O = ODD; AO = ADHD + ODD; C = controls. (T = Tukey, G = Games-Howell).

a Only significant differences (*p* < .05) are noted.

b Calendar days.

### Annual Incomes (€) at Age 20 and 30 Years (Years 2006 and 2016) in the Study Groups, Males, and Females

Data on the annual incomes for the study groups in the years 2006 (20-year follow-up) and 2016 (30-year follow-up) are presented in Table 4 for males. In the 2016 follow-up, when participants were 30 years old, the control group had a higher income (*M* = €30,875, *SD* = €20,326) than both the ADHD group (*M* = €27,341, *SD* = €15,998) and the ADHD + ODD group (*M* = €24,922, *SD* = €16,323; *p* < .05). The ADHD + ODD group had the lowest income among the study groups. No statistically significant differences were found between the ADHD and the ADHD + ODD group, or between the ODD and the control group in post hoc comparisons. In the 2006 follow-up, when participants were 20 years old, the ADHD group had a higher income (*M* = €11,306, *SD* = €10,051) than the control group (*M* = €9,394, *SD* = €8,987; *p* < .05).

**Table 4.** Annual Incomes (€) at Age 20 and 30 Years (Years 2006 and 2016) in the ADHD, ODD, ADHD + ODD, and Control Groups for Males and Females.

	ADHD	ODD	ADHD + ODD	Controls	Anova/Welch	post hoc test^a^ (T = Tukey, G = Games-Howell)
	*M* (*SD*)	*M* (*SD*)	*M* (*SD*)	*M* (*SD*)		
Males						
Age 20 years	11,306 (10,051)	9,912 (8,910)	9,619 (9,593)	9,394 (8,987)	*p* = .021	C < A *(T)*
Age 30 years	27,341 (15,998)	26,961 (18,998)	24,922 (16,323)	30,875 (20,326)	*p* < .001	C > A, C > AO *(T)*
Females						
Age 20 years	10,789 (7,223)	10,686 (7,505)	9,757 (7,554)	10,064 (11,999)	*p* = .879	—
Age 30 years	19,949 (11,365)	21,399 (12,147)	18,793 (10,950)	23,013 (14,804)	*p* < .001	C > A, C > AO *(T)*

*Note.* A = ADHD; O = ODD; AO = ADHD + ODD; C = controls. (T = Tukey, G = Games-Howell).

a Only significant differences (*p* < .05) are noted.

Data on the annual incomes of females in the study groups are also shown in Table 4. In the 30-year follow-up, the control group had a higher income (*M* = €23,013, *SD* = €14,804) than both the ADHD group (*M* = €19,949, *SD* = €11,365) and the ADHD + ODD group (*M* = €18,793, *SD* = €10,950; *p* < .05). The ADHD + ODD group had the lowest income among the study groups. No statistically significant differences were found between the ADHD and the ADHD + ODD group, or between the ODD and the control group in post hoc comparisons.

Results of the regression model for incomes in the study groups for males are presented in Table 5. In the unadjusted model, the ADHD group had 11.75% lower incomes compared to the controls, with a 95% CI [−17.22, −5.73]. The ADHD + ODD group had 13.50% lower incomes than the controls, with a 95% CI [−21.34, −4.97]. The ODD group did not reach statistical significance compared to the control group. Statistically significant results remained for the male ADHD group after adjusting for each confounding factor separately (adjusted models 1–4) and for the ADHD + ODD group when the model was adjusted for the educational attainments of the parents of the participants (model 3) and after adjusting for the family type of the participants (model 4). When all the confounding factors were used together, the results did not remain statistically significant for any of the symptomatic groups (adjusted model 5).

**Table 5.** Annual Incomes (€) at Age 30 (2016), OLS Regression With Dependent Variable in Natural Logarithmic Form, Presented in Exact Percentage Terms, Males, and Females.^a^

	Unadjusted		Adjusted 1		Adjusted 2		Adjusted 3		Adjusted 4		Adjusted 5	
	%	95 % CI	%	95 % CI	%	95 % CI	%	95 % CI	%	95 % CI	%	95 % CI
Males												
ADHD	**−11.75**	**[−17.22 to −5.73]**	**−7.23**	**[−13.15 to −1.00]**	**−9.34**	**[−14.79 to −3.54]**	**−10.42**	**[−16.05 to −4.40]**	**−11.04**	**[−16.81 to −4.97]**	−4.30	[−10.42–2.12]
ODD	−0.60	[−13.58–14.34]	1.41	[−11.40–16.07]	0.70	[−11.49–14.45]	−0.60	[−13.93–14.80]	−2.47	[−15.89–13.77]	1.31	[−11.57–3.77]
ADHD + ODD	**−13.50**	**[−21.34 to −4.97]**	−8.24	[−16.47–0.90]	−8.33	[−16.22–0.30]	**−13.32**	**[−21.26 to −4.78]**	**−13.58**	**[−21.49 to −4.88]**	−5.26	[−13.41–3.77]
Females												
ADHD	**−8.97**	**[−15.72 to −1.78]**	−1.98	[−8.70–5.34]	−7.23	[−14.02–0.10]	**−7.60**	**[−14.27 to −0.40]**	**−8.70**	**[−15.38 to −1.49]**	−0.10	[−6.95–7.36]
ODD	−5.82	[−15.13–4.60]	−2.37	[−11.04–7.14]	−4.21	[−13.58–6.08]	−5.82	[−15.21–4.60]	−5.45	[−15.04–5.23]	−1.59	[−10.69–8.33]
ADHD + ODD	**−10.95**	**[−19.27 to −1.78]**	−0.80	[−9.70–8.98]	**−9.34**	**[−17.63 to −0.20]**	−6.85	[−15.72 to −2.86]	**−10.60**	**[−19.10 to −1.39]**	3.56	[−6.01–14.11]

*Note*. Adjusted model 1 was adjusted for the educational attainment by the age of 32. Adjusted model 2 was adjusted for the psychiatric disorders other than ADHD or ODD (yes or no). Adjusted model 3 was adjusted for the educational attainments of the parents of the participants. Adjusted model 4 was adjusted for the family type. Adjusted model 5 was adjusted for the educational attainment by the age of 32, the educational attainments of the parents, family type, and psychiatric disorders other than ADHD or ODD. (To obtain the income gaps across different groups in exact percentage terms, we took the antilog (to base e) of the coefficients of the dummy variables indicating the groups, subtracted 1, and multiplied the differences by 100 (Gujarati, 2011, p. 55).)

a Statistically significant figures (*p* < .05) are in bold type.

Results of the regression model for annual incomes in the study groups for females are also presented in Table 5. In the unadjusted model, the ADHD group had 8.97% lower incomes compared to controls, with a 95% CI [−15.72, −1.78], and the number for the ADHD + ODD group was 10.95% with a 95% CI [−19.27, −1.78]. The ODD group did not reach statistical significance compared to the controls. Statistically significant results remained for the female ADHD group after adjusting for the educational attainments of the parents of the participants (model 3) and after adjusting for the family type of the participants (model 4). Also, statistically significant results remained for the female ADHD + ODD group after adjusting for the psychiatric disorders other than ADHD or ODD (model 2) and after adjusting for the family type of the participants (model 4). When all the confounding factors were used together, results did not remain statistically significant for any of the symptomatic groups (model 5).

### The Role of ADHD Symptom Clusters in the Main Results

For additional analysis, continuous measures of the inattentive and hyperactive-impulsive symptom clusters were used to elucidate regression analyses for annual incomes, with the dependent variable expressed in natural logarithmic form (see Supplemental Table 1). The inattentive symptom cluster exhibited the strongest, and negative relation to annual incomes. Hyperactive-impulsive symptom cluster was associated positively with annual incomes. Pearson correlation coefficient between continuous measures of the inattentive and hyperactive-impulsive symptom clusters was around .73 for both males and females. Results remained statistically significant when adjusted for the educational attainment by the age of 32 years, the educational attainments of the parents, family type, and psychiatric disorders other than ADHD or ODD.

## Discussion

To the best of our knowledge, this is the first population-based study including ADHD, ODD, and ADHD + ODD groups for both sexes to examine their associations with occupational outcomes and incomes. The findings suggested that the co-occurrence of ODD symptoms with ADHD symptoms in adolescence predicted the greatest deficits in occupational outcomes and incomes in adulthood for both males and females. Symptoms of ADHD and ADHD + ODD were associated with higher unemployment, more sick days, and lower annual incomes compared to the control group for both sexes. Furthermore, symptoms of ODD were associated with higher unemployment and more sick days for males, but they did not reach statistical significance in their association with annual incomes in this study. Annual income analyses within the study groups were adjusted for the educational level of the participants’ parents, the educational level of the participants themselves, family type, and psychiatric disorders other than ADHD or ODD. The results retained statistical significance when individual confounding factors were analyzed separately. However, when the models were adjusted for all confounding effects simultaneously, the income differences between the studied groups no longer appeared to be statistically significant. This probably reflects two related matters. First, the confounding variables are strongly interrelated, which may result in multicollinearity issues in estimation (e.g., estimated standard errors tend to increase as a result). Especially, education is highly statistically significantly dependent on parents’ education and is also associated with other psychiatric disorders (see Supplemental Table 3). Second, it is quite intuitive that having ADHD or ODD per se shouldn’t markedly affect an individual’s income or other labor market outcomes directly, but the effect likely comes indirectly through factors such as education.

Our results are consistent with previous studies suggesting an association between symptoms of ADHD and unemployment in adulthood. We also found that symptoms of ADHD in adolescence were associated with higher unemployment in young adulthood at ages 20 to 24 years, although this association is not clearly established in the literature. A clinical-based follow-up study in the US found that only 8% of young adults with ADHD were unemployed, which was not significantly different from the control group (Mannuzza et al., 1997). It is known that young adults with a history of ADHD tend to seek employment rather than pursue additional education after graduating from high school (Kuriyan et al., 2013). However, young adults with ADHD symptoms usually attain lower-status employment compared to their peers without ADHD symptoms (Biederman et al., 2008). Furthermore, we found that symptoms of ODD in adolescence were associated with higher unemployment in adulthood (ages 25–33 years) for males compared to controls. This association was not statistically significant in young adulthood (ages 20–24 years). This is a consistent finding with a population-based study from the US, which reported that symptoms of ODD were not associated with employment status in young adulthood (ages 22–29 years; B. J. Leadbeater & Ames, 2017). As no other studies have examined the relationship between ODD symptoms and employment status before, more research is needed.

Sick leave data was acquired from the registers of the Social Insurance Institution and Finnish Center for Pensions, covering information on all workers in Finland. The availability of sick leave is consistent across the country, irrespective of residence or employment status. In our sample, the control group had fewer sick days than all the symptomatic groups, which is consistent with a cross-sectional study of 414 clinically diagnosed adult ADHD patients, where symptoms of ADHD were associated with an increased likelihood of taking sick leave (Halmøy et al., 2009). No other previous studies have examined the relationship between symptoms of ODD and sick leave. Previous studies among the NFBC1986 have suggested that symptoms of ADHD and ODD are associated with substance use disorder, different types of injuries and an increased risk of undergoing psychiatric hospitalization during one’s lifetime (Hurtig et al., 2016; Mustonen et al., 2023; Nordström et al., 2013), all of which are likely causes of increased sick leave from work.

When observing the annual incomes of both males and females at 30 years of age, the results showed that the ADHD + ODD group had the lowest annual incomes among the study groups. A surprising result was found in the 20-year follow-up, where the male ADHD group had a higher income than the control group. Similar trends were noted for females but did not reach statistical significance. As pointed out in the literature, young adults with ADHD symptoms tend to enter the labor market more quickly than their peers without ADHD symptoms (Kuriyan et al., 2013), potentially explaining these results. Individuals without ADHD typically delay entering the workforce at 20 years of age, as they often pursue higher education. Supporting this, an American study reported that individuals with hyperactive symptoms in childhood had higher incomes than controls at 20 years of age (Fischer & Barkley, 2006). Another study noted that only 15% of the participants in the hyperactive group were full-time students at 20 years of age, while the control group had a similar rate of 66% (Barkley et al., 2006). The results from the NFBC1986 study sample have shown similar findings regarding the educational status (Seppä et al., 2023). In Finland, higher education studies (university or university of applied sciences) typically begin at around 19 years of age, after graduating from upper secondary school.

When examining the regression model for annual incomes in the study groups, we introduced the confounding factors one at a time into the model. When confounding factors related to family (educational attainments of the parents of the participants and the family type of the participants) were included, the results remained statistically significant in the adjusted model. Family background is known to be a significant factor in individuals’ occupational outcomes (Fletcher, 2014; Knapp et al., 2011; Vergunst et al., 2019). A follow-up study of 920 boys from the US found that family adversity, which included parents’ educational level and family structure (intact vs. non-intact), was associated with lower earnings in their analysis (Vergunst et al., 2019). In our study, fewer participants in the ADHD and ADHD + ODD groups lived with both of their biological parents during their childhood compared to the control group. Considering this adversity as a confounder in the regression analysis for the study groups, our results remained statistically significant for the ADHD and ADHD + ODD groups for both males and females. Furthermore, participants in the control group had parents with a higher educational attainment than those in the ADHD group in this sample. Educational attainment tends to be passed down from parents to their children and can, therefore, affect occupational outcomes. Results from a British birth cohort study suggested that workers whose mothers had higher educational attainment were estimated to earn more (Knapp et al., 2011). Even after adjusting for the educational level of the participants’ parents, our study showed that the male ADHD and ADHD + ODD groups and the female ADHD group were associated with lower incomes at 30 years of age compared to controls.

Furthermore, when the analyses were adjusted for the educational attainment of the participants by the age of 32, the results remained statistically significant for the male ADHD group. Educational attainment has been known to be a significant predictor of occupational outcomes. Results of a cross-sectional study from Norway suggested that educational attainment was a robust factor predicting the likelihood of not being in work, that is, the lower the educational level, the higher the risk of being out of work (Halmøy et al., 2009). The role of educational attainment has also been considered in population-based studies, where it has been suggested that educational attainment partially explains associations between ADHD and occupational outcomes (Fletcher, 2014; Jangmo et al., 2021; Rajah et al., 2023).

Psychiatric comorbidity with ADHD is more common than the exception, and previous studies suggest that psychiatric disorders such as anxiety, depression, and bipolar disorder alongside ADHD tend to worsen patients’ occupational outcomes (Halmøy et al., 2009; Soendergaard et al., 2015). A European clinically referred sample of adult ADHD patients reported that patients with ADHD and lifetime comorbid psychiatric disorders were more frequently unemployed than individuals with ADHD alone (Sobanski et al., 2007). A clinical-based study from Norway (*n* = 250) suggested that a higher number of comorbid mental disorders, especially anxiety disorder, with ADHD, were significantly related to long-term work disability for both sexes (Fredriksen et al., 2014). Our results regarding annual incomes between the study groups remained statistically significant for the ADHD group (males) and for the ADHD + DD group (females) compared to controls even after adjusting for other psychiatric disorders.

For more in-depth analyses, we explored ADHD presentations, including continuous measures of the inattentive and hyperactive-impulsive symptom clusters (see Supplemental Tables 1 and 2). In conclusion, the connection between adolescent ADHD symptoms and subsequent annual incomes was predominantly influenced by inattentive symptoms of ADHD, which is a consistent finding with previous studies (Fredriksen et al., 2014; Jangmo et al., 2021; Vergunst et al., 2019). Interestingly, the hyperactive-impulsive symptom cluster showed a positive association with annual incomes. While most studies have not found a link between hyperactive-impulsive symptom presentation and incomes (Barkley et al., 2006; Vergunst et al., 2019), Fischer & Barkley (2006) reported that individuals with hyperactive symptoms in childhood earned higher incomes than controls by the age of 20. It is possible that higher levels of motor activity, especially when linked to attentive behavior, could be beneficial for individuals with ADHD. For example, a recent study by Chan et al. (2023) suggests that increased hyperactivity is associated with greater resilience in children with ADHD alongside other factors, such as social skills and academic performance. Additionally, recent evidence from clinical trials on physical exercise shows positive links between increased movement levels and executive functions in children and adolescents with ADHD (Huang et al., 2023). Several other studies, including experimental research and within-subject designs, support this idea, suggesting that gross motor activity may enhance working memory and task performance in individuals with ADHD (Kofler et al., 2020; Sarver et al., 2015).

We also considered the potential presence of multicollinearity as an explanation for the results presented in Supplemental Table S1. Both pairwise correlation coefficients and Variance Inflation Factors (VIF) are commonly used tools for diagnosing multicollinearity (Schober & Schwarte, 2018; Vatcheva & Lee, 2016). In Supplemental Table 1, the VIF values range between 2.11 and 2.32. These values are not alarming based on conventional thresholds, but Vatcheva and Lee (2016) demonstrate that VIF less than 5 (VIF < 5) does not always indicate low multicollinearity. They note that even cutoffs of 0.5 for correlation coefficients are used in empirical research, but the most typical cutoff is 0.80. Dormann et al. (2013) improve understanding of collinearity and its management, suggesting |*r*| < .7 as a useful threshold for identifying distortion in model estimation. Supplemental Table 2 shows correlation coefficient values around .73 between inattentive and hyperactive-impulsive symptom clusters. It is plausible that participants scoring relatively high in the inattentive symptom cluster are also generally overrepresented among those scoring higher in the hyperactive-impulsive symptom cluster (even though they may not necessarily reach the cut-off points), which might explain the relatively high correlation (*r* > .70). Therefore, the findings presented in the Supplemental Material regarding ADHD symptom presentations should be interpreted with caution. Further research is needed to corroborate and expand upon the findings presented here.

Our study is consistent with previous studies suggesting that individuals with ADHD symptoms have lower incomes compared to controls (Altszuler et al., 2016; Fletcher, 2014; Jangmo et al., 2021). This association remained statistically significant even after adjustments. In contrast, the association between symptoms of ODD and annual incomes was not statistically significant compared to the control group. This is consistent with previous studies suggesting that disruptive behavior in childhood (hyperactivity, opposition, and aggression) is not associated with earnings in adulthood (Alatupa et al., 2013; Vergunst et al., 2019). ADHD is a neurodevelopmental disorder that involves deficits in executive functions, which are cognitive processes critical for the execution of goal-directed behavior (Castellanos et al., 2006). Executive dysfunction has been shown to be an important component of the early neuropsychology of ADHD rather than ODD (Brocki et al., 2007). Conversely, temperament, and anger proneness in particular, have been shown to play an important role in the development of externalizing behavior problems and, subsequently, ODD symptoms (Christensen & Baker, 2021). These reasons can explain why symptoms of ADHD predict worse occupational outcomes and lower incomes than ODD symptoms.

### Strengths and Limitations

The strength of this study stems from its use of a large, unselected, population-based sample comprising over 6,000 participants. This substantial sample size contributes statistical power in the analysis and enabled the consideration of various confounding factors and psychiatric comorbidities.

The study’s outcomes were derived from nationwide register data, ensuring reliability, with a minimal attrition rate. There are also limitations. This study had a relatively short follow-up time for incomes. Many individuals are expected to continue developing in their careers into their early forties, and this could potentially impact their earnings. The global generalizability of this study is limited due to differences in educational and occupational opportunities outside Finland. When comparing Finland to the US, it is important to note that Finland offers free education for all and provides more equal opportunities for career development. This study relied on subjective reports for symptoms of ADHD and ODD at ages 15 to 16 years. In most cases, it was either one of the parents who filled out the questionnaires, typically mothers. Although SWAN cut-offs suggest a likely diagnosis, they do not encompass a comprehensive psychiatric clinical assessment. Rating scales, like the SWAN, are cost-effective and valuable tools for assessing general psychopathology and its impact on social functioning. Moreover, the psychopathology associated with ADHD and ODD often intersects with various other psychiatric conditions, such as personality disorders, anxiety, and mood disorders (Kooij et al., 2012). These conditions may coexist as comorbidities in patients with ADHD or ODD, and their symptoms can mimic those of ADHD or ODD, including hyperactivity, impulsivity, inattention, and disruptive behavior. This complexity further complicates the challenges of recognizing and diagnosing ADHD and ODD. Nevertheless, the SWAN comprehensively measures the entire spectrum of behavior rather than focusing solely on pathological symptoms and signs associated with ADHD or ODD.

## Conclusions

Our findings suggested that symptoms of ADHD were associated with adverse occupational outcomes and lower incomes compared to controls. This association was primarily driven by the presence of inattentive symptoms of ADHD. ODD symptoms, on their own, were associated with occupational outcomes but not with incomes. However, the presence of ODD symptoms exacerbated the issues, leading to the ADHD + ODD group displaying the most significant deficits among all the study groups compared to the controls. In a clinical context, early recognition of ADHD and ODD symptoms is important due to their long-term impact. Implementing effective student counseling in schools becomes crucial for guiding adolescents in making informed decisions about their academic, vocational, artistic, or athletic pursuits. Additionally, acknowledging the substantial influence of parents and the family environment is essential in shaping an individual’s path from adolescence to adulthood and influencing their career development.

## Supplemental Material

sj-docx-1-jad-10.1177_10870547241259329 – Supplemental material for Associations of Symptoms of ADHD and Oppositional Defiant Disorder (ODD) in Adolescence With Occupational Outcomes and Incomes in Adulthood Supplemental material, sj-docx-1-jad-10.1177_10870547241259329 for Associations of Symptoms of ADHD and Oppositional Defiant Disorder (ODD) in Adolescence With Occupational Outcomes and Incomes in Adulthood by Sampo Seppä, Sanna Huikari, Marko Korhonen, Tanja Nordström, Tuula Hurtig and Anu-Helmi Halt in Journal of Attention Disorders
