# Supplementary material for: Associations of Symptoms of ADHD and Oppositional Defiant Disorder (ODD) in Adolescence With Occupational Outcomes and Incomes in Adulthood
Source: J Atten Disord. 2024 Jun 12;28(10):1392–405. doi: 10.1177/10870547241259329 (PMC11292981; doi:10.1177/10870547241259329)
Supplement: sj-docx-1-jad-10.1177_10870547241259329 – Supplemental material for Associations of Symptoms of ADHD and Oppositional Defiant Disorder (ODD) in Adolescence With Occupational Outcomes and Incomes in Adulthood [file sj-docx-1-jad-10.1177_10870547241259329.docx]

***Table S1. Annual incomes at age 30 (2016), OLS regression with dependent variable in natural logarithmic form, presented in exact percentage terms, continuous measures of the inattentive and hyperactive-impulsive symptom clusters.***

|  | **Unadjusted** | |  | **Adjusted** | |  |
| --- | --- | --- | --- | --- | --- | --- |
|  | **coeff.** | **95 % CI** | **VIF** | **coeff.** | **95 % CI** | **VIF** |
| **Males** |  |  |  |  |  |  |
| **Inattentive** | **-9.07** | **-11.26–-6.83** | **2.18** | **-5.36** | **-7.71–**  **-2.95** | **2.28** |
| **Hyperactive-**  **Impulsive** | **4.27** | **1.90–6.70** | **2.18** | **3.11** | **0.77–5.50** | **2.14** |
| **Females** |  |  |  |  |  |  |
| **Inattentive** | **-12.60** | **-14.57–-10.58** | **2.11** | **-8.27** | **-10.44–-6.05** | **2.32** |
| **Hyperactive-**  **Impulsive** | **6.89** | **4.56–9.28** | **2.11** | **6.05** | **1.14–8.44** | **2.15** |
|  |  |  |  |  |  |  |

Statistically significant figures (*p*<0.05) are in bold type. VIF = variance inflation factor.

In unadjusted model, the natural logarithm of annual income in 2016 were regressed against both continuous measures of the Inattentive and Hyperactive/Impulsive symptom clusters.

Adjusted model were adjusted for the educational attainment by the age of 32, the educational attainments of the parents, family type, and psychiatric disorders other than ADHD or ODD.

***Table S2. Pearson correlation coefficients between continuous measures of the Inattentive and Hyperactive/Impulsive symptom clusters***

|  | ***Inattentive*** | ***Hyperactive/***  ***impulsive*** |
| --- | --- | --- |
| ***Males*** |  |  |
| ***Inattentive*** | 1.000 |  |
| ***Hyperactive/impulsive*** | 0.732 | 1.000 |
| ***Females*** |  |  |
| ***Inattentive*** | 1.000 |  |
| ***Hyperactive/impulsive*** | 0.729 | 1.000 |

All correlations were statistically significant (*p*<0.05).

***Table S3. Results of the Pearson χ2 test of independence between educational attainments of the subjects at age 32 years and other confounders.***

|  | **Educational attainments of the subjects at age 32 years** | | | | |
| --- | --- | --- | --- | --- | --- |
|  | ***Comprehensive school*** | ***Upper secondary school*** | ***Institution of higher education*** | ***Total*** | ***χ2(df),***  ***p-value*** |
| **Males** |  |  |  |  |  |
| **Educational attainments of the subjects´s parents** |  |  |  |  | 142.24 (4), *p*<0.001 |
| Comprehensive school | 66 (30.70) | 121 (56.38) | 28 (13.02) | 215 |  |
| Upper secondary school | 427 (25.89) | 1,005 (60.95) | 217 (13.16) | 1,649 |  |
| Institution of higher education | 211 (18.07) | 602 (51.54) | 355 (30.39) | 1,168 |  |
| **Family type of the subjects** |  |  |  |  | 39.46 (2), *p*<0.001 |
| Both parents | 141 (6.09) | 1,215 (52.44) | 961 (41.48) | 2,317 |  |
| Others | 72 (10.86) | 394 (59.43) | 197 (29.71) | 663 |  |
| **Psychiatric disorders of the subjects*** |  |  |  |  | 117.30 (2), *p*<0.001 |
| Yes | 89 (16.04) | 340 (61.26) | 126 (22.70) | 555 |  |
| No | 141 (5.52) | 1,348 (52.76) | 1,066 (41.72) | 2,555 |  |
| **Females** |  |  |  |  |  |
| **Educational attainments of the subjects´s parents** |  |  |  |  | 115.35 (4), *p*<0.001 |
| Comprehensive school | 28 (3.87) | 348 (48.13) | 347 (47.99) | 723 |  |
| Upper secondary school | 48 (2.74) | 727 (41.45) | 979 (55.82) | 1,754 |  |
| Institution of higher education | 9 (1.60) | 122 (21.63) | 433 (76.77) | 564 |  |
| **Family type of the subjects** |  |  |  |  | 50.83 (2), *p*<0.001 |
| Both parents | 49 (2.13) | 843 (36.59) | 1,412 (61.28) | 2,304 |  |
| Others | 32 (4.61) | 337 (48.56) | 325 (46.83) | 694 |  |
| **Psychiatric disorders of the subjects*** |  |  |  |  | 103.74 (2), *p*<0.001 |
| Yes | 46 (6.26) | 369 (50.20) | 320 (43.54) | 735 |  |
| No | 41 (1.73) | 855 (36.03) | 1,477 (62.24) | 2,373 |  |
